# Women's experiences in the transition to menopause: a qualitative research

**DOI:** 10.1186/s12905-022-01633-0

**Published:** 2022-02-26

**Authors:** Mansoureh Refaei, Soraya Mardanpour, Seyedeh Zahra Masoumi, Parisa Parsa

**Affiliations:** 1 Department of Mother and Child Health, Mother and Child Care Research Center, School of Nursing and Midwifery, Hamadan University of Medical Sciences, Hamadan, Iran; 2 Department of Midwifery and Reproductive Health, School of Nursing and Midwifery, Hamadan University of Medical Sciences, Hamadan, Iran; 3 Department of Midwifery and Reproductive Health, Mother and Child Care Research Center, School of Nursing and Midwifery, Hamadan University of Medical Sciences, Hamadan, Iran

**Keywords:** Experiences, Fear, Hope, Premenopause, Qualitative research, Viewpoint

## Abstract

**Background:**

Around the time of transition to menopausal period, women experience mental, and psychological disorders that require adequate attention to these symptoms. This study aimed to explore the experiences of women in the face of premenopausal symptoms.

**Methods:**

This qualitative study was conducted using a content analysis method in Javanrood, Iran, in 2020. The data were collected through in-depth semi-structured face to face interviews with 16 premenopausal using interview guide in a private room in comprehensive health centers. The women inclusion criteria were approaching menopause, having irregular menstruation, and having no disease or medication that affects menstruation. Furthermore, the exclusion criteria were the absence of menstruation for more than 12 months, and the women's refusal to continue the interview. The participants were selected using purposive sampling and sampling continued until data saturation. The collected data were analyzed with MAXQDA10 software following the multi-step method proposed by Graneheim and Lundman.

**Results:**

The participants' mean age was 47 ± 2.98 years. The data analysis revealed 5 categories including: "menopause and aging", "life transformation", confrontation of fear and hope", "life adjustment", and "need to facilitate the transition time".

**Conclusion:**

This study suggested the women's experience of the transition to menopause was characterized by the fear of the future and its consequences and the need for reassurance about it. Besides, the women sought solutions to their problems in health care providers, peers, and the family.

## Background

A woman's reproductive period is divided into three stages, including the reproductive stage, the transition to menopause, and the postmenopausal stage. The transition to menopause begins at the onset of menstrual irregularities and continues until the last menstrual cycle [1]. The transition to menopause is characterized by a gradual change from premenopause to menopause [2] marking one of the most critical stages in a woman's life in middle age [3, 4]. The average age of menopause in Iran is 48.26 years with a range of 46.85 to 49.67 years [5]. It has been estimated that about 17 million of the population of Iran will be women aged 40–60 by 2036 [6]. Therefore, a large number of women will be about to undergo menopause and will experience this event.

Women experience new symptoms during menopause. These include increased variability during the menstrual cycle, vasomotor changes [7], decreased sexual function [2], urinary problems [8], mood swings [9], physical and mental fatigue associated with impaired memory, decreased concentration, and amnesia [10].

Women have different experiences around menopause and after it due to physiological and psychological changes [11]. Experience is the knowledge or mastery of an event or subject gained through engagement or exposure [12]. Not all women experience and respond to menopause in the same way [13]. Besides, their subjective experiences of menopause may not be the same [11]. These new experiences can affect women's lives [14].

Understanding women's concerns by providing evidence can help enrich existing studies and assist in designing effective support strategies, planning training programs, and developing effective infrastructure for them. An awareness of the experience of women in dealing with premenopausal symptoms and identifying their problems and needs can help health policymakers and managers to provide the best care for middle-aged women so that they go through this period with minimum complications. Most studies on the changes and problems of women have focused on the postmenopausal period. More studies are needed on healthy women who are going through this period to identify positive and negative changes in them [13].

The present study aimed to explain the experiences of women in the face of changes in the transition to menopause. Qualitative research is useful for understanding and describing human experiences, perceptions, and feelings as this method can provide researchers with a deep insight into the perceptions and experiences of individuals [15]. Accordingly, a qualitative content analysis technique was employed in the present study.

## Methods

### Design and participants

This content analysis study was performed in the comprehensive health centers of Javanrood city, Kermanshah province, Iran, in 2020. The research population included all premenopausal women who had referred to health centers in Javanrood to receive health care. The inclusion criteria were being over 45 years old, approaching menopause based on family history, irregular menstruation that was not due to illness, and the willingness to express the physical or psychological changes that were considered to be due to approaching menopause. The exclusion criterion was having any disease that could affect menopausal symptoms, such as cardiovascular disease, endocrine disorders, mental disorders, and a history of hysterectomy or oophorectomy. The participants were selected using purposive sampling with maximum diversity in terms of age, education, marital life, and social class, and the sampling process continued until data saturation. The participants were 16 women who were experiencing premenopausal period.

### Sampling methods

The data were collected through semi-structured face to face in-depth interviews with the participants using open-ended questions. After obtaining the necessary permits and making arrangements to attend health centers, the researcher provided some explanations about the study to the women who met the inclusion criteria and if they wished, the interview was conducted individually in a room in the health center away from the noise. No one was present in the interview room except the participants and the researcher. The interviewees' voices were recorded with their consent. The duration of the interviews ranged from 15 to 30 min. No one refused to participate in the study. Two participants were interviewed twice. The second time the telephone interview was conducted.

The initial questions asked in the interviews were as follows:

1. What physical and mental changes are you experiencing around menopause?
2. Do you feel that you are experiencing physical or mental changes related to the premenopausal period?
3. What is your understanding of the physical and psychological changes?
4. What problems have these changes created in your daily life?
5. What physical and mental needs do you feel concerning these changes?
6. What are you doing to cope with these changes?

Some probing questions were used to obtain more information and to clarify the content of interviews, such as: can you explain more? The collected data were analyzed using conventional content analysis following the method proposed by Graneheim and Lundman [16]. After each interview, the content of the interview was typed word for word in Microsoft Word. Each interview was considered as a unit of analysis. To gain a general understanding of the transcripts, they were read carefully several times. Then, the transcripts were divided into meaning units and after condensing each meaning unit, they were labeled as code. The codes were classified into sub-subcategories based on their similarities and differences. Finally, the related subcategories were extracted according to the latest concepts in the transcript and by classifying them, the categories were identified (Table 1):

**Table 1:** An example of data analysis

Meaning unit	Code	Sub-subcategory	Subcategory	Category
My menopausal habits have become irregular. My body gets hot to some extent. I sweat a lot. Then my skin dries up a bit. I feel very tired. My energy has decreased a lot compared to the last few years. I feel old. My knees hurt a lot	Menstrual changes	Physical changes	Undergoing changes	Mixing menopause and aging
	Sweating and hot flashes			
	Dry and wrinkled skin			
	Lack of energy			
	Physical pain			

To evaluate the trustworthiness of the data in this study, the four criteria proposed by Lincoln and Guba were used [17]. The credibility of the data was ensured by the researchers' involvement in the subject under study for a long time and allocating sufficient time for collecting the data. Since the researcher had several years of experience working in a midwifery office, she had a good interaction with the women and using her professional experience, she gained the trust of the participants to be able to collect real, enriched data on the research problem by taking into account different perspectives. Also researcher conducted member check for credibility. Besides, the conformability of the findings was ensured via peer check (having the data reviewed by the colleagues). The dependability or consistency of the data was confirmed via external check (having the data reviewed by experienced experts) and also via the code–recode strategy that was used in the data analysis process. Finally, to improve the transferability of the data, rich, accurate, and step-by-step descriptions were used.

## Results

The participants in this qualitative study were 16 women with a mean age of 47.5 ± 2.28 years. Besides, 43.75% of the participants had more than diploma education and most of them (81.25%) were unemployed. Moreover, 81.25% of the participants lived with their husband, and 68.75% of them reported a moderate-income level (Table 2):

**Table 2:** The participants' demographic characteristics

Variable	Mean ± SD	Frequency/number
Age (years)	47.5 ± 2.28	
Number of children	2.5 ± 1.31	
Duration of menopausal changes (month)	7.5 ± 2.5	
Number of participants		16
*Education*		
Diploma		3
Less than diploma		6
More than diploma		7
*Employment*		
Employed		3
Unemployed		13
*Economic status*		
Poor		1
Moderate		11
High		3
Excellent		1
*Living with the husband*		
Yes		12
No		4

### Codes, sub- subcategories, subcategories, and categories identified in the study

The data analysis revealed 388 primary codes, 24 sub-subcategories, 11 subcategories, and 5 categories as displayed in Table 3.

**Table 3:** Categories, subcategories, and sub-subcategories

Sub-subcategories	Subcategories	Categories
Physical changes Psychological changes Sexual changes	Feeling changes	Mixing menopause and aging
Aging causes changes Menopause causes changes	The ambiguity of the cause of changes	
Bad temperateness Feeling deficient Intellectual conflict Apathy	Negative mood/behavioral reactions	Life change
Changing relationships with spouse Changing relationships with children	Negative effects of changes on social life	
Exhaustion of power and ability Fear of persistence of symptoms	Concerns about the persistence/intensification of symptoms	Confrontation of fear and hope
Seeing oneself competent Hope for recovery	Self-consolation	
Seeing a doctor and consultant Getting rid of disturbing thoughts and problems	Attempting to overcome problems	Life adjustment
Feeling better physically Having a better mood	Effective efforts	
Spouse attention Decreased spouse attention	The need to be understood by the spouse	Needs to facilitate the transition time
Having good friends Sharing experiences	The need for sympathetic peers	
Needing to see a doctor Needing to see a consultant	The need for specialized guidance	

#### Category 1: Mixing menopause and aging

The results of the study indicated that the participants' experiences of premenopause were characterized by gradual exposure to new changes that were taking place with unknown causes.

*Feeling changes:* All participants pointed to physical, psychological, and sexual changes including menopausal changes, skin changes, sweating and hot flashes, body aches, lack of energy, sleep disorders, impatience, forgetfulness, depression, decreased libido, and decreased sexual intercourse:

My menopausal cycle has become irregular. My body gets somehow hot. I sweat a lot. Then my skin dries up a bit ... I feel very tired. My energy has decreased a lot compared to the last few years. I feel old and I feel pain in my knees (Participant 16, 48 years old).

*The ambiguity of the cause of changes:* Some participants attributed these changes to the onset of menopause and some to aging and the onset of aging:

I'm feeling changes in my body that are new to me. For example, now I feel hot and very tired. I always like to be alone. ... My husband is complaining about it. He says my temperament has changed. I don't know whether it's due to menstruation or aging (Participant 11, 51 years old).

Some of the participants mostly considered aging as the cause of changes in themselves. These were the people who experienced the most physical symptoms such as body aches and dryness:

My lack of energy has made me think I'm not young anymore. I consider my impatience to be due to my age. I think I'm old enough to feel old. When I wake up in the morning and have to massage myself a little to relax my body. I have not been feeling like this before. This feeling started about five or six months ago. At first, it was not intense, but it got gradually worse (Participant 3, 48 years old).

#### Category 2: Life change

The analysis of the participants' experiences indicated negative behavioral and mood reactions that were caused by the changes in them, affecting their relationships with their husbands and children.

*Negative mood/behavioral reactions:* Most participants reported behavioral changes, including bad-temperedness, most of which were due to physical changes in themselves. They stated that they experienced changes such as irritability, anger, aggression, low mood, discomfort, mental conflict, and apathy:

I cannot sleep comfortably at night. When I wake up in the morning, I feel tired and upset. When I look at other women, I feel that I'm missing something compared to them. It makes me feel bad and inefficient. I'm feeling old, less energetic, lazy, and unmotivated (Participant 2, 45 years old).

*The negative effect of changes on social life:* These problems have had negative and adverse effects on the participants' life, so that the person's family relationships, i.e. relationships with spouse and children have also been negatively affected:

My husband is unhappy and says I don't behave like before ... he gets cold little by little and comes home late, he mostly entertains himself with the housework, he thinks I don't like him (Participant 1, 49 years old).

I feel bored and tired. I feel depressed, aggressive and restless at home. The children also complain a little about the way I behave (Participant 16, 50 years old).

#### Category 3: Confrontation of fear and hope

Given the changes that have taken place and the consequences and impact on the participants' life, they stated that they were facing an internal conflict, which was the confrontation of fear and hope.

*Fear of persistence and aggravation of symptoms:* Following the changes, the women stated that they were struggling with disturbing thoughts about their disabilities and getting old quickly. The cause was not important to them, but the consequence, that is, old age and disability, was highlighted by them. Furthermore, they were afraid that these changes would remain stable and get worse.

These changes have had a profound effect on my psyche and mental health. The problems I did not have before but now have got worse. I am afraid they will continue getting worse. I don't know what I should do (Participant 4, 41 years old).

*Self-consolation:* In addition to the fear of aggravation of symptoms and disabilities, the participants reported that they also experienced a sense of hope. In fact, there is still hope that the situation is temporary and may end after a while:

I feel inadequate and less energetic. I give hope to myself that these will be temporary and over and I will feel well again. This situation will end for better or worse (Participant 5, 47 years old).

#### Category 4: Life adjustment

The participants stated that in the face of these recent changes, they were trying to overcome the problems, and the efforts made by them were somewhat effective.

*Trying to overcome problems:* Most of the participants stated that they were trying to overcome the problems faced by them:

I have to talk with someone to help me control myself so that I do not worry my husband and children. I want them to know that I can cope with this stage as I did with many other events (Participant 7, 47 years old).

*Effective efforts:* Most of the participants stated that they were trying to reduce the problems by engaging in activities and hobbies such as exercising, going out with friends, shopping, etc., or reduce the problems by seeing a consultant and doctor and following the recommendations provided by them. Accordingly, they succeeded in reducing the problems to some extent by doing such activities.

#### Category 5: Needs to facilitate the transition time

Women undergoing menopausal changes have needs including receiving more attention and support from their husbands and being understood by them. Besides, they need friends to share experiences and also spend time with them, and they also need awareness and advice to have a better lifestyle and find answers to their questions.

*The need to be understood the husband:* Some participants were concerned that because of these changes and the lack of awareness and understanding of their husband, their husband may pay less attention to them and consider them as a woman who is in the process of aging:

I need my husband to understand me. Sometimes I think he is looking at other women and when I look closely, I see that they are almost a few years younger than me and I envy them. I would like my husband to know what happened is a part of every woman's life and it will happen to them sooner or later (Participant 4, 41 years old).

*The need for empathetic peers:* The participants reported that they need to have peer friends who are in a similar situation so that in addition to sharing their experiences and ideas, they can spend time and empathize with them.

I would like to have a few friends like myself to consult and sympathize with them. I like to spend time with them and talk to them easily about the problems and this common pain; think and sympathize about it together (Participant 14, 48 years old).

*Need for expert advice:* Women at this age need to be aware of menopausal changes and how to adapt to them. In fact, they need to make sure whether these problems are permanent or just typical of the current period:

I need more information and must see a doctor who can answer all my questions and help me not grow old or these things happen later. I need advice and information so that I can undergo this period more easily and without further injury to my body (Participant 9, 43 years old).

## Discussion

The present study explored the experiences of women facing the transition to menopause in Iran. This study showed that premenopausal women are experiencing changes recently faced them. Hakimi et al. 2018 showed that the most common physical symptoms experienced by postmenopausal women are musculoskeletal problems and the most common psychological symptoms are mood swings and irritability [18]. Menopause is considered the "beginning of a new phase of life" and undergoing experiences such as "changes in physical and mental health" [19].

The participants in the present study reported the reasons for these changes as aging or approaching menopause but it was unclear to them from which these changes really came. Sakdiah et al. (2015) showed that Malay women identified physical changes, including diminished ability, aging, decreased health and physical, hormonal and emotional changes, as signs of aging, and most of them associated menopause with aging [20]. Nosek et al. (2010) showed that a more negative attitude towards aging and a more positive attitude towards menopause affect menopausal symptoms [21]. Postmenopausal women have a more positive attitude towards menopause and aging than women around menopause. Providing useful information about menopause and aging may help reinforce a positive attitude toward menopause in women [11]. Therefore, people must have a positive attitude towards these two phenomena. By clearing this ambiguity for women, their attitudes toward menopause and aging can be changed, and this positive attitude may alleviate the symptoms associated with this period.

During this period, women experience problems in social life, especially in terms of family relationships, and relationships with their husband and children. During menopause, sexual incompatibility occurs between couples [22]. Women who experience menopause are more vulnerable to marital problems [23]. Satisfaction with the husband and children affects the menopausal symptoms [18]. The present study showed that the behavior of the husband and children is a reflection of the behavioral changes in the woman during premenopausal periode. If the husband and children are familiar with the psychological changes of women during the transition to menopause and provide the necessary support, the mutual satisfaction of the women can help reduce menopausal symptoms and improve the experience of women during this period.

The analysis of the participants' experiences suggested that women face fear and hope together. The women showed concern about the persistence or exacerbation of menopausal symptoms and on the other hand hope for improvement of symptoms. Women who have just started experiencing and are gradually undergoing menopausal symptoms and changes are worried about the future and persistence of their symptoms, and those whose symptoms have diminished are hoping for improvement. Women think of menopause as a sign of loss of youth, and vitality, and as an indicator of the end of life and the beginning of old age and disability, and this causes sadness and fear in them. Actually, they fear aging and disability, not menopause. Hakimi et al. (2014) suggested that women who have recently reached menopause have concerns in four areas: isolation, health, aging, and disability [14]. In contrast to Hakimi's study, the women in the present study did not report isolation or fear of health problems, probably because they were still experiencing the transition to menopause. Accordingly, it can be argued that women's experiences in the transition to menopause are different from their experiences during menopause, thus, their concerns and issues in these two periods need to be addressed separately.

Following the problems and the changes that women experience during premenopause, women seek solutions to manage the existing situation. Most of the participants stated that they saw a doctor and counselor to solve the problems upon the advice of their husband and children. During menopause, women adopt coping strategies to enhance their physical and emotional health [24]. Ghazanfarpour et al. (2018) suggested that one of the strategies adopted by women for sexual problems created during menopause is to get help from their peers and refer to health care providers [22]. Therefore, health care providers must acquire sufficient skills to be able to help couples in this period. Wong et al. (2018) suggested that knowledge, financial support, and family understanding are important to help women manage menopausal symptoms [23].

Furthermore, women in the menopausal cycle need their husbands to understand them, and they need knowledge and also someone who can answer their questions. Besides, they need friends who have experienced a similar situation to empathize with them. An important point revealed in the present study is the role of peers and the need for women to consult and sympathize with women who are experiencing menopausal symptoms themselves, which has not been addressed in most studies [25]. Considering the role of peers, effective strategies can be adopted to reduce the problems of women in this period. Cognitive-behavioral group therapies are effective in reducing mental health problems during the climacteric period [26]. Women need to be prepared for menopause. Personal health care tailored to individual needs, preferences, and expectations should be provided to them [24]. Wong et al. (2018) stated that knowledge, financial support, and family understanding are important to help women manage menopause [23].

The present study explored the experiences of women during the transition to menopause, while most studies have examined women in the postmenopausal or menopausal period. Moreover, researchers have tried to provide rich, accurate, and step-by-step descriptions and document all stages of the research to make the data more transferable and decrease non-generalizability that is specific to qualitative studies.

## Conclusion

Fear of the future and its consequences and the need to be confident about it are significant experiences of women during the transition to menopause. Women seek solutions to these problems in their health care providers, families, and peers. Therefore, in addition to having full awareness of the course of menopause, health care providers should familiarize women with this process. Furthermore, the role of peers and family should also be taken into account when planning for this group of women. For further research, the effect of psychological interventions in the treatment of these disorders in postmenopausal women is recommended.

## Data Availability

The datasets used and/or analyzed in relation to the current study are avail- able from the corresponding author upon reasonable request.
